# Sample size and power calculations in Mendelian randomization with a single instrumental variable and a binary outcome

**DOI:** 10.1093/ije/dyu005

**Published:** 2014-03-06

**Authors:** Stephen Burgess

**Affiliations:** Department of Public Health and Primary Care, University of Cambridge

**Keywords:** Mendelian randomization, sample size, power, binary outcome, allele score

## Abstract

**Background:** Sample size calculations are an important tool for planning epidemiological studies. Large sample sizes are often required in Mendelian randomization investigations.

**Methods and results:** Resources are provided for investigators to perform sample size and power calculations for Mendelian randomization with a binary outcome. We initially provide formulae for the continuous outcome case, and then analogous formulae for the binary outcome case. The formulae are valid for a single instrumental variable, which may be a single genetic variant or an allele score comprising multiple variants. Graphs are provided to give the required sample size for 80% power for given values of the causal effect of the risk factor on the outcome and of the squared correlation between the risk factor and instrumental variable. R code and an online calculator tool are made available for calculating the sample size needed for a chosen power level given these parameters, as well as the power given the chosen sample size and these parameters.

**Conclusions:** The sample size required for a given power of Mendelian randomization investigation depends greatly on the proportion of variance in the risk factor explained by the instrumental variable. The inclusion of multiple variants into an allele score to explain more of the variance in the risk factor will improve power, however care must be taken not to introduce bias by the inclusion of invalid variants.

**Key Messages**

- Resources are provided for investigators to perform sample size and power calculations for Mendelian randomization with a binary outcome.
- The sample size required for a given power level is greater with a binary outcome than a continuous outcome, and is highly dependent on the proportion of the variance in the risk factor explained by the instrumental variable.

## Introduction

Sample size calculations are an important part of experimental design. They inform an investigator of the expected power of a given analysis to reject the null hypothesis. If the power of an analysis is low, then not only is the probability of rejecting the null hypothesis low, but when the null hypothesis is rejected, the posterior probability that the rejection of the null hypothesis is not simply a chance finding is low.^1^

Mendelian randomization is the use of genetic variants as instrumental variables for assessing the causal effect of a risk factor on an outcome from observational data.^2^ Genetic variants are chosen which are specifically associated with a risk factor of interest, and not associated with variables which may be confounders of the association between the risk factor and outcome.^3^ Such a variant divides the population into groups which are similar to treatment arms in a randomized controlled trial.^4^ Under the instrumental variable assumptions,^5^^,^^6^ a statistical association between the genetic variant and the outcome implies that the risk factor has a causal effect on the outcome.^7^ However, as genetic variants typically explain a small proportion of the variance in risk factors, the power to detect a significant association between the variant and outcome in an applied Mendelian randomization context can be low.^8^ Sample size analysis is particularly important to inform whether a null finding is representative of a true null causal relationship, or simply a lack of power to detect an effect size of clinical interest.

Sample size calculations have been previously presented for Mendelian randomization experiments with continuous outcomes. Calculations based on asymptotic statistical theory have been presented with a single instrumental variable (IV), whether that IV is a single genetic variant or an allele score.^9^ An allele score (also called a genetic risk score) is a single variable summarizing multiple genetic variants as a weighted or unweighted sum of risk factor-increasing alleles.^10^ A simulation study for estimating power has also been presented with both single and multiple IVs.^11^ These approaches have shown good agreement. However, in many cases, the outcome in a Mendelian randomization experiment is binary (dichotomous), such as disease. In this paper, we present power calculations for Mendelian randomization studies with a binary outcome. We assume the context of a case-control study where the causal parameter of interest is an odds ratio, although the calculations are also valid for other study designs.

## Methods and Results

We give results for the asymptotic variance of IV estimators with a single IV, and for the resulting sample size needed in a Mendelian randomization study to obtain a given power level. We initially present formulae with a continuous outcome (this reviews material previously covered by Freeman *et al.*^9^) and then analogous formulae with a binary outcome. We concentrate on estimates from the ratio (or Wald) method, as this method makes few parametric assumptions, relying only on a linear relationship between the conditional expectation of the outcome (or in the binary case, the logistic function of the probability of the outcome) and the risk factor.^12^ If the imprecision in the estimate of the genetic association with the risk factor is negligible, then estimates of power and sample size from the ratio method also correspond to those from assessment of the causal relationship of the risk factor on the outcome by testing the association between the genetic variant and outcome.

Other estimation approaches are possible with a binary outcome^13^ but these either give equivalent estimates to the ratio method with a single IV (the two-stage predictor substitution method^14^) or are not recommended for general use in applied practice. These include the two-stage residual inclusion method, due to inconsistency for a parameter with a natural interpretation,^15^ and the generalized method of moments (GMM) and structural mean models (SMM) methods, due to potential lack of identifiability of the causal parameter (S Burgess *et al.*, unpublished data).

### Power with a continuous outcome

With a single IV and a continuous outcome, the IV estimates from the ratio, two-stage least squares (2SLS) and limited information maximum likelihood (LIML) methods coincide.^16^ The estimator can be expressed as the ratio between the coefficient from the regression of the outcome (Y) on the genetic variant (G), divided by the coefficient from the regression of the risk factor (X) on the variant:
(1)
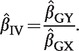

The asymptotic variance of this IV estimator is given by the formula:
(2)
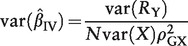

where *R*_Y_ = *Y* – β_1_. *X* is the residual of the outcome on subtraction of the causal effect of the risk factor, and 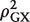 is the square of the correlation between the risk factor *X* and the IV *G*.^17^ The coefficient of determination (*R*^2^) in the regression of the risk factor on the IV is an estimate of 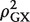. The IV in these calculations could either be a single genetic variant or an allele score.^10^

The asymptotic variance of the conventional regression (ordinary least squares, OLS) estimator of the association between the risk factor *X* and the outcome *Y* is given by the formula:
(3)
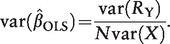

The sample size necessary for an IV analysis to demonstrate a non-zero association for a given magnitude of causal effect is therefore approximately equal to that for a conventional epidemiological analysis to demonstrate the same magnitude of association divided by the 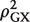 value for the IV.^18^ If the significance level is *a* and the power desired to test the null hypothesis is 1−β, then the sample size required to test a causal effect of size β_1_ using IV analysis is:^9^
(4)
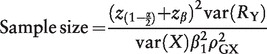

where *z* is a quantile function, so that *z*_α_ is the 100*α* percentile point on the standard normal distribution. If the significance level is 0.05 and the power is 0.8, then the sample size to test for a change of β_1_ standard deviations in *Y* per standard deviation increase in *X* is:
(5)
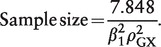

For a given sample size N, the power to detect a causal effect (in the same direction the true effect) can be calculated as:
(6)
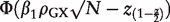

where Φ is the cumulative distribution function of the standard normal distribution. This is the inverse function of the quantile function (Φ(z*_α_*) = *α*).

**Figure 1.**
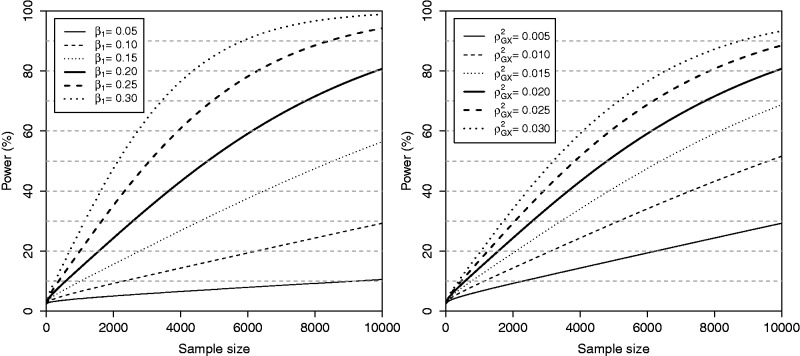
Power curves varying the sample size with continuous outcome and a single instrumental variable. Left panel: for a fixed value of the IV strength 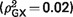 and different values of the size of the causal effect (*β*_1_ = 0.05, 0.1, … , 0.3). Right panel: for a fixed value of the causal effect (*β*_1_ = 0.2) and varying the size of the IV strength 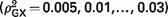

We use these formulae to construct power curves for Mendelian randomization using a significance level of 0.05. In Figure 1 (left), we fix the squared correlation 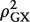 at 0.02, meaning the variant explains on average 2% of the variance of the risk factor, and vary the size of the effect β_1_ = 0.05, 0.1, 0.15, 0.2, 0.25, 0.3 and the sample size *N* = 1000 to 10 000. In Figure 1 (right), we fix the size of the effect at β_1_ = 0.2 and vary the squared correlation 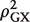 = 0.005, 0.01, 0.015, 0.02, 0.025, 0.03 and the sample size as before. In each of the figures, the power to detect a positive causal relationship is displayed; this tends to 0.025 as the sample size tends to zero. We see that the power increases as the causal effect increases, and as the IV explains more of the variance in the risk factor (the 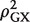 parameter or the expected value of the *R*^2^ statistic increases).

Similar formulae to these have been made available in an online tool for calculating either power for a given sample size or sample size needed for a given power, taking the causal effect (β_1_) and squared correlation (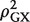) parameters, as well as the variance of the risk factor and outcome, and the observational (OLS) coefficient of the risk factor from regression on the outcome.^19^

### Power with a binary outcome

With a single IV and a binary outcome, the same IV estimator^1^ as in the continuous outcome case can be evaluated, except that a logistic model is typically used in the regression of the outcome on the genetic variant.^12^ The asymptotic variance of this estimator can be approximated using the delta method for the ratio of two estimates.^20^ The leading term in the expansion is:
(7)
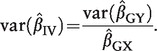

Although further terms from the delta method could be included, these are usually much smaller in magnitude. In the simulation example later in the paper, if the association between the risk factor and IV is estimated using data on the entire sample of control participants, the second and third terms in the expansion are two orders of magnitude smaller than the leading term (Figure 1). The asymptotic variance of the coefficient 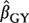 from logistic regression is:
(8)
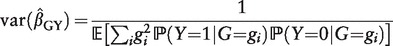

where *i* indexes individuals. This expression is obtained by differentiation of the log-likelihood. If the probability of an event does not depend greatly on the value of the genetic IV, then 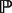
(Y = 1 | G = g_i_) ≈ 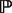
(Y = 1) which is the ratio of cases to participants in the sample. This approximation will be reasonable if the genetic variant does not explain a large proportion of the variance in the risk factor, and/or the effect of the risk factor on the outcome is not extreme. We assume (without loss of generality) that the mean of *G* is 0 and the variance is 1, so that 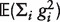 = *N*, where *N* is the sample size. The square of the coefficient 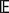 is approximately equal to var(*X*) 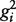. This gives:
(9)
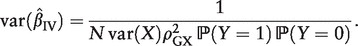

The sample size required to detect an effect of size β_1_ per standard deviation increase in *X* for 80% power with a significance level of 0.05 is therefore
(10)
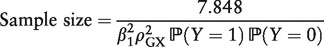

where the effect β_1_ is a log odds ratio. If there are to be an equal number of cases and controls, 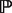
(Y = 1) = 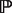
(Y = 0) = 0.5, and:
(11)
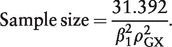

The corresponding power to detect a causal effect of size β_1_ with a significance level of 0.05 is:
(12)
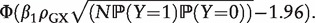

Similar power curves to Figure 1 in the binary outcome setting are given in the Web Appendix (available as Supplementary data at *IJE* online).

**Figure 2.**
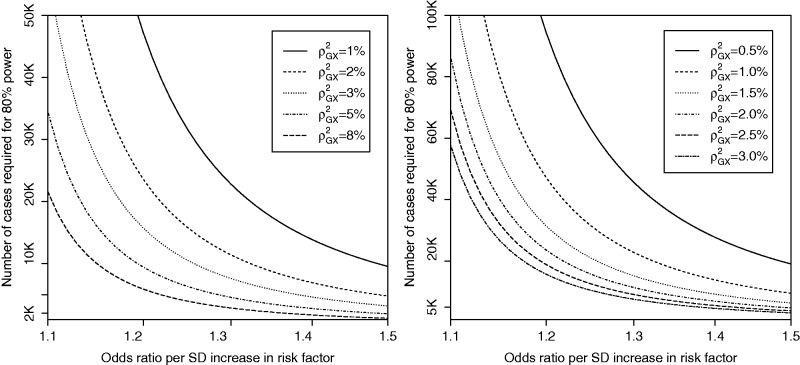
Number of cases required in a Mendelian randomization analysis with a binary outcome and a single instrumental variable for 80% power with a 5% significance level and 1:1 ratio of cases:controls varying the size of causal effect [odds ratio per standard deviation (SD) increase in risk factor, exp(*β*_1_)] for different values of IV strength. Left panel: 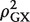 = 1%–8%. Right panel: 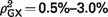 = 0.5%–3.0%

We use these approximations to calculate the number of cases needed to obtain 80% power in a Mendelian randomization analysis with a binary outcome for different values of β_1_ and 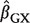, assuming a 1:1 ratio of cases to controls. The results are displayed in Figure 2. We note that when the genetic variants explain a small proportion of the variance in the risk factor, large sample sizes are required to detect even moderately large causal effects with reasonable power.

An R^21^ script for performing sample size and power calculations is provided in the Appendix (available as Supplementary data at *IJE* online). This code enables the calculation of the sample size required for a chosen power level given the values of β_1_ and 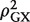, as well as the power given the values of β_1_, 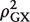 and the chosen sample size. A calculator using this code is available online.^22^

### Validation simulation

In order to validate the estimates of sample size and power, we simulate data on a genetic variant, a continuous risk factor and an outcome. The data-generating model for individuals indexed by *i* is:
(13)
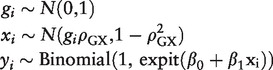

where expit(*x*) = (exp(*x*) / 1 + exp(*x*)) is the inverse of the logit function and β_1_ is the log odds ratio per unit (which here equals 1 standard deviation) increase in the risk factor. The genetic variant is modelled by a standard normal distribution; it can be regarded as a standardized weighted allele score. The parametric relationship between *X*, *G* and ρ_GX_ ensures that the proportion of variance in the risk factor explained by the instrumental variable in a large sample is 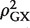. We also simulate data with a dichotomous risk factor; details are given in the Web Appendix (available as Supplementary data at *IJE* online).

We set β_0_ = −3 so that the outcome has a prevalence of about 5% in the population from which the case-control sample is taken. We take three values of β_1_ = 0.1, 0.2, 0.3, three values of 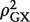 = 0.01, 0.02, 0.03, three sample sizes (10 000, 20 000, and 30 000 cases), and two values of the ratio of cases to controls (1:1 and 1:2). For each set of parameter values, we calculate the estimate of the power from equation (12) using a significance level of 0.05, and compare this with the number of times the 95% confidence interval for the ratio estimate excludes the null based on 10 000 simulated datasets.

The 95% confidence interval for the ratio method used in calculating the power of the simulation method is constructed using Fieller’s method,^23^ and so does not rely on the same asymptotic assumption as the analytical method for estimating the power. Previous simulations have shown that confidence intervals from Fieller’s method maintain nominal coverage levels even with weak instruments.^16^ To obtain a case-control sample of the necessary size, we initially simulate data for a large number of individuals, and then take the required number of cases and controls from this population.

### Simulation results

Results from the validation simulation are given in Table 1. The Monte Carlo standard error (the expected variation from the true value due to the limited number of simulations) in the simulation estimates of power is at most 0.5%. The coverage levels of the 95% confidence interval from Fieller’s method are close to 95% throughout (between 94.8 and 95.9 for the 54 scenarios).

**Table 1.** Validation simulation to compare estimates of power in a Mendelian randomization analysis with a continuous risk factor and a binary outcome from analytical formula and simulation study with a 5% significance level varying the size of causal effect (*β*_1_), the IV strength (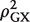), the sample size and the ratio of cases to controls

Case:control ratio = 1:1		10000 cases		20000 cases		30000 cases	
		Formula	Simulation	Formula	Simulation	Formula	Simulation
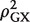 = 0.01	β_1_ = 0.1	10.5%	10.2%	16.9%	16.6%	23.1%	22.4%
	β_1_ = 0.2	29.3%	28.4%	51.6%	51.2%	68.8%	69.5%
	β_1_ = 0.3	56.4%	56.4%	85.1%	85.0%	95.7%	95.7%
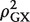 = 0.02	β_1_ = 0.1	16.9%	17.2%	29.3%	28.9%	41.0%	41.1%
	β_1_ = 0.2	51.6%	51.0%	80.7%	80.2%	93.4%	93.6%
	β_1_ = 0.3	85.1%	84.9%	98.9%	98.9%	99.9%	100.0%
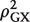 = 0.03	β_1_ = 0.1	23.1%	22.9%	41.0%	40.8%	56.4%	57.0%
	β_1_ = 0.2	68.8%	68.5%	93.4%	93.3%	98.9%	99.0%
	β_1_ = 0.3	95.7%	95.5%	99.9%	99.9%	100.0%	100.0%

Case:control ratio = 1:2		10000 cases		20000 cases		30000 cases	
		Formula	Simulation	Formula	Simulation	Formula	Simulation
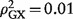 = 0.01	β_1_ = 0.1	12.6%	12.9%	21.0%	21.4%	29.3%	28.9%
	β_1_ = 0.2	37.2%	37.5%	63.7%	64.4%	80.7%	81.1%
	β_1_ = 0.3	68.8%	68.2%	93.4%	93.3%	98.9%	98.8%
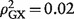 = 0.02	β_1_ = 0.1	21.0%	21.2%	37.2%	37.8%	51.6%	51.6%
	β_1_ = 0.2	63.7%	63.9%	90.4%	90.7%	97.9%	97.9%
	β_1_ = 0.3	93.4%	93.2%	99.8%	99.8%	100.0%	100.0%
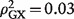 = 0.03	β_1_ = 0.1	29.3%	29.0%	51.6%	51.4%	68.8%	68.8%
	β_1_ = 0.2	80.7%	80.8%	97.9%	97.7%	99.8%	99.9%
	β_1_ = 0.3	98.9%	98.9%	100.0%	100.0%	100.0%	100.0%

We note that estimates of power from the formula of equation (12) are similar to those from the simulation approach. There is no apparent systematic bias in the estimates from the analytical formula, with simulation estimates being greater and less than those from the formula a similar number of times (when rounded to nearest 0.1%, the estimate from the simulation was less 24 times and greater 19 times). Estimates from both approaches are no more different than would be expected due to chance alone. Similar results are obtained with a dichotomous risk factor; details are given in Web Table A1 (available as Supplementary data at *IJE* online). In comparing estimates of power with equal numbers of cases, greater power is achieved when there is a case:control ratio of 1:2 than with a ratio of 1:1. However, when the total sample size is fixed, the estimate of power is greatest when the numbers of cases and controls are equal. This can be seen by comparing estimates with 30 000 cases and a ratio of 1:1, and with 20 000 cases and a ratio of 1:2.

In response to concerns from a reviewer that the power estimates may not be valid with a discrete instrumental variable (such as a single nucleotide polymorphism) or when there is confounding, additional validation simulations were performed in these scenarios. Results are given in the Web Appendix (Web Tables A2–A4, available as Supplementary data at *IJE* online). No substantial differences were observed from the validation simulation in the main paper when the instrumental variable was discrete. When there was confounding, estimates from the analytical formula slightly overestimated power, particularly when the confounding was in the same direction as the causal effect. However, this overestimation was slight (on average less than 1% when the confounding was in the opposite direction, and less than 2% when the confounding was in the same direction). As the magnitude of confounding is not possible to estimate in applied practice, conservative estimates of the correlation and causal effect parameters used in power calculations are recommended, particularly if confounding is thought to be substantial.

## Discussion

In this paper, we have provided information on sample sizes and power calculations in a Mendelian randomization analysis with a single IV and a binary outcome. We have shown in the continuous setting how the power depends on the magnitude of causal effect and the proportion of variance in the risk factor explained by the IV. With a binary outcome, the precision of the coefficient in the regression of the outcome on the IV is reduced compared with a continuous outcome, as the outcome can only take two values. As a result, the required sample sizes to obtain 80% power are much larger.

For a given applied example, the magnitude of the causal effect of a risk factor is fixed, as is the expected proportion of variance in the risk factor explained by each variant. However, the expected proportion of variance in the risk factor explained by the IV depends on the choice of IV. The required sample size for a given power level can be reduced (or equivalently the expected power at a given sample size can be increased) by including more genetic variants into the IV. This can be achieved by using multiple variants as separate IVs,^13^ or as a single IV using an allele score approach. With an allele score, power can be further increased by the use of relevant weights for the variants.^10^ Provided that weights are not derived naively from the data under analysis, the allele score approach avoids some of the problems of bias from weak instruments resulting from using many IVs.^24^ A disadvantage of the inclusion of many variants in an IV analysis, whether in a multiple IV or an allele score model, is that one or more of the variants may not be a valid IV. If a variant is associated with a confounder of the risk factor–outcome association, or with the outcome through a pathway not via the risk factor of interest, then the estimate associated with this IV may be biased. If the function and relevance of some variants as IVs are uncertain, investigators will have to balance the risk of a biased analysis against the risk of an underpowered analysis. Sensitivity analysis may be a valuable tool to assess the homogeneity of IV estimates using different sets of variants.

If there are missing data, this may adversely impact the power of an analysis. When there are multiple genetic variants, individuals with sporadic missing genetic data can be included in an analysis using an imputation approach.^25^ This can minimize the impact of missing data on the power of the analysis, particularly if the distributions of genetic variants are correlated (the variants are in linkage disequilibrium).

The calculations in this paper make several assumptions. The distribution of the IV estimator is assumed to be well approximated by a normal distribution. This is known to be a poor approximation when the IV is weak;^26^ however, if the IV is weak, then the power will usually be low. The standard deviation of this normal distribution is assumed to be close to the first-order term from the delta expansion. This term only involves the uncertainty in the coefficient from the genetic association with the outcome. The uncertainty in the estimate of the genetic association with the risk factor is not accounted for. Typically, this uncertainty will be small in comparison as the genetic association with the outcome is assumed to be mediated through the risk factor. Again, if this uncertainty is large, then the power of the analysis will usually be low. If a more precise estimate of the power is required, either further terms from the delta expansion could be used, or a direct simulation approach could be undertaken. The model of the logistic-transformed probability of an outcome event is assumed to be linear in the risk factor. As the power is very sensitive to the squared correlation term 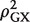, it is advisable to take a conservative estimate of this parameter, or to perform a sensitivity analysis for a range of values of 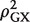. Despite these approximations, the validation simulation suggests that estimates of sample size and power from the formulae in this paper will be close to the true values for a range of realistic values of the parameters involved.

The ratio method used in this paper has been criticized for use with binary outcomes to estimate an odds ratio.^27^^,^^28^ This is due to the non-collapsibility of the odds ratio, meaning that the parameter estimate depends on the choice of covariate adjustment.^29^ This is a general property of odds ratios, and not a specific feature of the ratio method. The estimate from the ratio method approximates a population averaged odds ratio,^15^ and is close to a conditional odds ratio under certain specific circumstances.^30^ The choice of odds ratio estimate does not affect the consistency of the estimator under the null.^31^ As effect estimation is usually secondary to the demonstration of a causal effect, the precise identification of the parameter estimated by the ratio method is not of particular importance in Mendelian randomization analyses, and over-literal interpretation of Mendelian randomization estimates should be avoided even outside the odds ratio case.^32^

Although the sample sizes required in Mendelian randomization experiments are often large, it is not always necessary to measure the risk factor on all of the participants in a study. Simulations have shown that, in some cases, 90% of the power of the complete-data analysis can be obtained while only measuring the risk factor for 10% of participants.^33^ This means that obtaining measurements of the risk factor, which may be expensive or impractical for a large sample, should not be the prohibitive factor for a Mendelian randomization investigation.

## Supplementary Data

Supplementary data are available at *IJE* online.

**Conflict of interest:** None declared.

Supplementary Data
