# Supplementary material for: Sample size and power calculations in Mendelian randomization with a single instrumental variable and a binary outcome
Source: Int J Epidemiol. 2014 Mar 6;43(3):922–9. doi: 10.1093/ije/dyu005 (PMC4052137; doi:10.1093/ije/dyu005)
Supplement: Supplementary Data [file supp_43_3_922__index.html]

Sample size and power calculations in Mendelian randomization with a single instrumental variable and a binary outcome — Supplementary Data 

# Sample size and power calculations in Mendelian randomization with a single instrumental variable and a binary outcome

## Supplementary Data

files

**Files in this Data Supplement:**

- Supplementary Data - pdf file
