# Supplementary material for: Sample size and power calculations in Mendelian randomization with a single instrumental variable and a binary outcome
Source: Int J Epidemiol. 2014 Mar 6;43(3):922–9. doi: 10.1093/ije/dyu005 (PMC4052137; doi:10.1093/ije/dyu005)
Supplement: Supplementary Data [file supp_dyu005_binpower050713app.pdf]

Web Appendix

A.1 Validation simulation with dichotomous risk factor

In this section, we repeat the validation simulation from the main paper, except that the risk factor variable is taken to be dichotomous. In order to simulate data on a dichotomous risk factor D, we initially simulate data for a continuous risk factor X as in the original generating model, and then dichotomize the variable at the value 0. We model the outcome Y as depending on the dichotomized value $D = 1_{\{X>0\}}$. If the outcome were to depend on D through the value of X, then the instrumental variable G would be an invalid IV for D, as there would be an alternative pathway from G to Y via X. The data-generating model is:

$$
\begin{aligned}
g_i &\sim \mathcal{N}(0, 1) \qquad (1)\\
x_i &\sim \mathcal{N}(g_i \rho_{GX}, 1 - \rho_{GX}^2)\\
d_i &= 1_{\{x_i>0\}}\\
y_i &\sim \text{Binomial}(1, \text{expit}(\beta_0 + \beta_1 d_i))
\end{aligned}
$$

where $1_{\{.\}}$ is an indicator function, taking value 1 if the subscripted condition is satisfied, and 0 otherwise.

The same parameters were used as in the simulation in the main body of the paper. Results for 10 000 simulated datasets are given in Web Table A1. As in the case with the continuous risk factor, estimates of the power from the analytic formula and the simulation study are similar throughout. Estimates of power are lower with the dichotomous risk factor than in the continuous case as the variance of the risk factor is lower. The coverage of the 95% confidence interval from Fieller's theorem is between 94.5 and 95.5 in the 54 scenarios.

Case:control ratio = 1:1		10 000 cases		20 000 cases		30 000 cases	
		Formula	Simulation	Formula	Simulation	Formula	Simulation
$\rho^2_{GX} = 0.01$	$\beta_1 = 0.1$	4.8%	4.9%	6.0%	6.4%	6.9%	7.1%
	$\beta_1 = 0.2$	8.7%	8.0%	11.5%	12.3%	16.4%	15.9%
	$\beta_1 = 0.3$	13.8%	13.2%	21.9%	22.1%	30.4%	31.3%
$\rho^2_{GX} = 0.02$	$\beta_1 = 0.1$	6.0%	5.8%	8.3%	7.8%	10.4%	10.3%
	$\beta_1 = 0.2$	11.8%	12.3%	19.8%	20.5%	28.5%	28.3%
	$\beta_1 = 0.3$	22.1%	21.8%	40.3%	39.5%	53.8%	53.2%
$\rho^2_{GX} = 0.03$	$\beta_1 = 0.1$	7.2%	6.9%	10.0%	10.5%	13.1%	13.2%
	$\beta_1 = 0.2$	16.4%	16.0%	27.8%	28.8%	39.9%	38.7%
	$\beta_1 = 0.3$	33.0%	31.1%	53.4%	54.1%	71.8%	70.3%
Case:control ratio = 1:2		10 000 cases		20 000 cases		30 000 cases	
		Formula	Simulation	Formula	Simulation	Formula	Simulation
$\rho^2_{GX} = 0.01$	$\beta_1 = 0.1$	5.0%	5.3%	6.9%	6.4%	8.0%	8.3%
	$\beta_1 = 0.2$	9.8%	9.2%	15.9%	14.5%	20.0%	20.2%
	$\beta_1 = 0.3$	16.1%	15.7%	27.9%	28.0%	38.8%	38.4%
$\rho^2_{GX} = 0.02$	$\beta_1 = 0.1$	6.6%	6.9%	9.4%	9.8%	12.0%	12.3%
	$\beta_1 = 0.2$	15.2%	14.6%	25.5%	25.0%	35.7%	35.1%
	$\beta_1 = 0.3$	28.7%	28.1%	47.2%	49.0%	68.3%	66.1%
$\rho^2_{GX} = 0.03$	$\beta_1 = 0.1$	8.1%	8.2%	12.0%	12.6%	16.5%	16.3%
	$\beta_1 = 0.2$	20.4%	20.0%	36.4%	34.8%	50.8%	49.7%
	$\beta_1 = 0.3$	38.5%	39.1%	63.1%	66.3%	84.1%	83.2%

Web Table A1: Validation simulation to compare estimates of power in a Mendelian randomization analysis with a dichotomous risk factor and a binary outcome from analytic formula and simulation study with a 5% significance level varying the size of causal effect (β_1), the IV strength (ρ^2_{GX}), the sample size and the ratio of cases to controls

A.2 Validation simulation with discrete instrumental variable

In response to concerns from a reviewer, we repeat the validation simulation from the main paper, except that the instrumental variable is taken to be discrete. In particular, we allow the instrumental variable to take 3 values (0, 1, 2) corresponding to a genetic variant which is a single nucleotide polymorphism, and the number of risk factor-increasing alleles. The minor allele frequency of the single nucleotide polymorphism is taken to be 0.3. The data-generating model is:

$$\begin{aligned} g_i &\sim \text{Binomial}(2, 0.3) \\ \tilde{x}_i &\sim \mathcal{N}(\alpha_1 g_i, 1) \\ x_i &= \frac{\tilde{x}_i}{\text{sd}(\tilde{X})} \\ y_i &\sim \text{Binomial}(1, \text{expit}(\beta_0 + \beta_1 x_i)) \end{aligned} \tag{2}$$

The variable $\tilde{X}$ is normalized by dividing by its standard deviation to ensure that the variance of the risk factor X is 1. The same parameters were used as in the simulation in the main body of the paper, except that the ρ^2_{GX} parameter is replaced by a α_1 parameter, representing the per allele effect of the genetic variant. Three values of this parameter were taken: $\alpha_1 = 0.2, 0.3, 0.4$. The value of ρ^2_{GX} in the power calculation is taken as the mean value of the R^2 statistic from the regression of X on G across simulations.

Results for 10 000 simulated datasets are given in Web Table A1. As in the case with the continuous instrumental variable, estimates of the power from the analytic formula and the simulation study are similar throughout.

Case:control ratio = 1:1		10 000 cases		20 000 cases		30 000 cases	
		Formula	Simulation	Formula	Simulation	Formula	Simulation
$\alpha_1 = 0.2$	$\beta_1 = 0.1$	14.7%	14.9%	25.1%	24.7%	35.0%	35.2%
	$\beta_1 = 0.2$	44.4%	44.9%	72.9%	74.0%	88.3%	88.5%
	$\beta_1 = 0.3$	77.8%	77.5%	97.1%	97.4%	99.7%	99.7%
$\alpha_1 = 0.3$	$\beta_1 = 0.1$	27.1%	26.6%	47.9%	47.4%	64.7%	65.1%
	$\beta_1 = 0.2$	77.0%	76.7%	96.8%	96.7%	99.7%	99.7%
	$\beta_1 = 0.3$	98.3%	98.1%	100.0%	100.0%	100.0%	100.0%
$\alpha_1 = 0.4$	$\beta_1 = 0.1$	42.7%	42.1%	70.8%	70.3%	86.7%	87.1%
	$\beta_1 = 0.2$	94.4%	94.6%	99.9%	99.9%	100.0%	100.0%
	$\beta_1 = 0.3$	100.0%	100.0%	100.0%	100.0%	100.0%	100.0%

Case:control ratio = 1:2		10 000 cases		20 000 cases		30 000 cases	
		Formula	Simulation	Formula	Simulation	Formula	Simulation
$\alpha_1 = 0.2$	$\beta_1 = 0.1$	18.2%	18.3%	31.7%	32.2%	44.4%	44.2%
	$\beta_1 = 0.2$	55.5%	54.3%	84.3%	84.7%	95.3%	95.4%
	$\beta_1 = 0.3$	88.2%	88.5%	99.3%	99.6%	100.0%	100.0%
$\alpha_1 = 0.3$	$\beta_1 = 0.1$	34.4%	34.6%	59.6%	59.3%	77.0%	77.5%
	$\beta_1 = 0.2$	87.6%	87.8%	99.3%	99.3%	100.0%	100.0%
	$\beta_1 = 0.3$	99.7%	99.7%	100.0%	100.0%	100.0%	100.0%
$\alpha_1 = 0.4$	$\beta_1 = 0.1$	53.5%	54.6%	82.5%	82.4%	94.4%	94.6%
	$\beta_1 = 0.2$	98.4%	98.6%	100.0%	100.0%	100.0%	100.0%
	$\beta_1 = 0.3$	100.0%	100.0%	100.0%	100.0%	100.0%	100.0%

Web Table A2: Validation simulation to compare estimates of power in a Mendelian randomization analysis with a discrete instrumental variable, continuous risk factor and a binary outcome from analytic formula and simulation study with a 5% significance level varying the size of causal effect (β_1), the IV strength (α_1), the sample size and the ratio of cases to controls

A.3 Validation simulation with confounding

In response to concerns from a reviewer, we repeat the validation simulation from the main paper, except allowing confounding in the data-generating model between the risk factor and outcome. The data-generating model is:

$$\begin{aligned}
g_i &\sim \mathcal{N}(0, 1) \\
u_i &\sim \mathcal{N}(0, 1) \\
x_i &\sim \mathcal{N}(g_i\rho_{GX} + 0.4u_i, 1 - 0.16 - \rho_{GX}^2) \\
y_i &\sim \text{Binomial}(1, \text{expit}(\beta_0 + \beta_1 x_i + \beta_2 u_i))
\end{aligned} \tag{3}$$

The same parameters were used as in the simulation in the main body of the paper. Two values were taken for $\beta_2 = \pm 0.6$, corresponding to positive (in the same direction as the causal effect) and negative (in the opposite direction to the causal effect) confounding scenarios.

Results for 10 000 simulated datasets are given in Web Table A3 (positive confounding) and Web Table A4 (negative confounding). Estimates of the power from the analytic formula are slightly higher than those from the simulation analysis in both positive and negative confounding scenarios, although the average differences between the power estimates were 1.5% (positive confounding) and 0.9% (negative confounding). In practice, the level of confounding is unknown, so conservative estimates of the correlation and causal effect parameters in power calculations are recommended. In their simulations, Pierce et al. did not find that power changed substantially when confounding was introduced into their simulations [11].

Case:control ratio = 1:1		10 000 cases		20 000 cases		30 000 cases	
		Formula	Simulation	Formula	Simulation	Formula	Simulation
$\rho^2_{GX} = 0.01$	$\beta_1 = 0.1$	10.5%	10.0%	16.9%	15.7%	23.1%	22.2%
	$\beta_1 = 0.2$	29.3%	27.9%	51.6%	49.9%	68.8%	66.0%
	$\beta_1 = 0.3$	56.4%	53.8%	85.1%	82.6%	95.7%	94.1%
$\rho^2_{GX} = 0.02$	$\beta_1 = 0.1$	16.9%	16.1%	29.3%	27.4%	41.0%	39.2%
	$\beta_1 = 0.2$	51.6%	48.4%	80.7%	79.0%	93.4%	92.2%
	$\beta_1 = 0.3$	85.1%	82.7%	98.9%	98.2%	99.9%	99.9%
$\rho^2_{GX} = 0.03$	$\beta_1 = 0.1$	23.1%	22.2%	41.0%	38.5%	56.4%	54.1%
	$\beta_1 = 0.2$	68.8%	65.7%	93.4%	92.2%	98.9%	98.6%
	$\beta_1 = 0.3$	95.7%	94.5%	99.9%	99.9%	100.0%	100.0%

Case:control ratio = 1:2		10 000 cases		20 000 cases		30 000 cases	
		Formula	Simulation	Formula	Simulation	Formula	Simulation
$\rho^2_{GX} = 0.01$	$\beta_1 = 0.1$	12.6%	11.9%	21.0%	20.2%	29.3%	27.8%
	$\beta_1 = 0.2$	37.2%	35.2%	63.7%	61.4%	80.7%	78.1%
	$\beta_1 = 0.3$	68.8%	65.9%	93.4%	91.6%	98.9%	98.8%
$\rho^2_{GX} = 0.02$	$\beta_1 = 0.1$	21.0%	19.7%	37.2%	34.7%	51.6%	49.3%
	$\beta_1 = 0.2$	63.7%	61.5%	90.4%	88.8%	97.9%	97.5%
	$\beta_1 = 0.3$	93.4%	92.3%	99.8%	99.8%	100.0%	100.0%
$\rho^2_{GX} = 0.03$	$\beta_1 = 0.1$	29.3%	27.9%	51.6%	48.8%	68.8%	66.3%
	$\beta_1 = 0.2$	80.7%	78.1%	97.9%	97.3%	99.8%	99.7%
	$\beta_1 = 0.3$	98.9%	98.7%	100.0%	100.0%	100.0%	100.0%

Web Table A3: Validation simulation to compare estimates of power in a Mendelian randomization analysis with a continuous risk factor and a binary outcome with positive confounding from analytic formula and simulation study with a 5% significance level varying the size of causal effect (β_1), the IV strength (ρ^2_{GX}), the sample size and the ratio of cases to controls

Case:control ratio = 1:1		10 000 cases		20 000 cases		30 000 cases	
		Formula	Simulation	Formula	Simulation	Formula	Simulation
$\rho^2_{GX} = 0.01$	$\beta_1 = 0.1$	10.5%	10.2%	16.9%	16.0%	23.1%	22.2%
	$\beta_1 = 0.2$	29.3%	28.2%	51.6%	51.0%	68.8%	67.5%
	$\beta_1 = 0.3$	56.4%	54.5%	85.1%	84.1%	95.7%	94.9%
$\rho^2_{GX} = 0.02$	$\beta_1 = 0.1$	16.9%	16.4%	29.3%	28.1%	41.0%	39.9%
	$\beta_1 = 0.2$	51.6%	50.1%	80.7%	79.8%	93.4%	92.8%
	$\beta_1 = 0.3$	85.1%	83.8%	98.9%	98.6%	99.9%	99.9%
$\rho^2_{GX} = 0.03$	$\beta_1 = 0.1$	23.1%	22.4%	41.0%	39.2%	56.4%	54.9%
	$\beta_1 = 0.2$	68.8%	66.8%	93.4%	92.5%	98.9%	98.7%
	$\beta_1 = 0.3$	95.7%	94.9%	99.9%	99.9%	100.0%	100.0%

Case:control ratio = 1:2		10 000 cases		20 000 cases		30 000 cases	
		Formula	Simulation	Formula	Simulation	Formula	Simulation
$\rho^2_{GX} = 0.01$	$\beta_1 = 0.1$	12.6%	12.5%	21.0%	19.7%	29.3%	28.0%
	$\beta_1 = 0.2$	37.2%	36.0%	63.7%	62.4%	80.7%	79.3%
	$\beta_1 = 0.3$	68.8%	67.2%	93.4%	92.5%	98.9%	98.5%
$\rho^2_{GX} = 0.02$	$\beta_1 = 0.1$	21.0%	21.2%	37.2%	35.0%	51.6%	49.8%
	$\beta_1 = 0.2$	63.7%	62.1%	90.4%	89.5%	97.9%	97.5%
	$\beta_1 = 0.3$	93.4%	92.8%	99.8%	99.7%	100.0%	100.0%
$\rho^2_{GX} = 0.03$	$\beta_1 = 0.1$	29.3%	29.2%	51.6%	49.2%	68.8%	67.4%
	$\beta_1 = 0.2$	80.7%	78.7%	97.9%	97.7%	99.8%	99.8%
	$\beta_1 = 0.3$	98.9%	98.9%	100.0%	100.0%	100.0%	100.0%

Web Table A4: Validation simulation to compare estimates of power in a Mendelian randomization analysis with a continuous risk factor and a binary outcome with negative confounding from analytic formula and simulation study with a 5% significance level varying the size of causal effect (β_1), the IV strength (ρ^2_{GX}), the sample size and the ratio of cases to controls

A.4 R code for performing sample size and power calculations

We here provide R code for performing sample size and power calculations. The code requires the proposed values of the causal effect (β_1, a log odds ratio) and squared correlation (ρ^2_{GX}), and can either provide the sample size (total number of cases and controls) required for a given level of power, or the power for a given sample size. A calculator implementing this code is available online [23].

```
expit <- function(x) { return(exp(x)/(1+exp(x))) }
rsq   = 0.02     # squared correlation
b1    = 0.2      # causal effect (log odds ratio per SD
b1    = log(1.2) #                or log of OR per SD)
sig   = 0.05     # significance level (alpha)
pow   = 0.8      # power level       (1-beta)
ratio = 1        # ratio of cases:controls = 1:ratio

cat("Sample size required for ", pow*100, "% power: ",
   (qnorm(1-sig/2)+qnorm(pow))^2/b1^2/rsq/(ratio/(1+ratio))/(1/(1+ratio)))

n = 40000  #  Sample size
cat("Power of analysis with ", n, "participants: ",
  pnorm(sqrt(n*rsq*(ratio/(1+ratio))*(1/(1+ratio)))*b1-qnorm(1-sig/2)))
```

A.5 Power curves with a binary outcome

We use the formulae from the paper to construct power curves for Mendelian randomization with a binary outcome using a significance level of 0.05. In Figure A1 (left), we fix the squared correlation ρ^2_{GX} at 0.02, meaning the variant explains on average 2% of the variance of the risk factor, and vary the size of the effect $\beta_1 = 0.05, 0.1, 0.15, 0.2, 0.25, 0.3$ and the sample size $N = 1000$ to 10 000. In Figure A1 (right), we fix the size of the effect at $\beta_1 = 0.2$ and vary the squared correlation $\rho^2_{GX} = 0.005, 0.01, 0.015, 0.02, 0.025, 0.03$ and the sample size as before. These parameters are the same as in Figure 1 with a continuous outcome. In each of the figures, the power to detect a positive causal relationship is displayed; this tends to 0.025 as the sample size tends to zero. The ratio of cases:controls is assumed to be 1:1.

These graphs are similar in shape to those with a continuous outcome, but the power is greatly reduced.

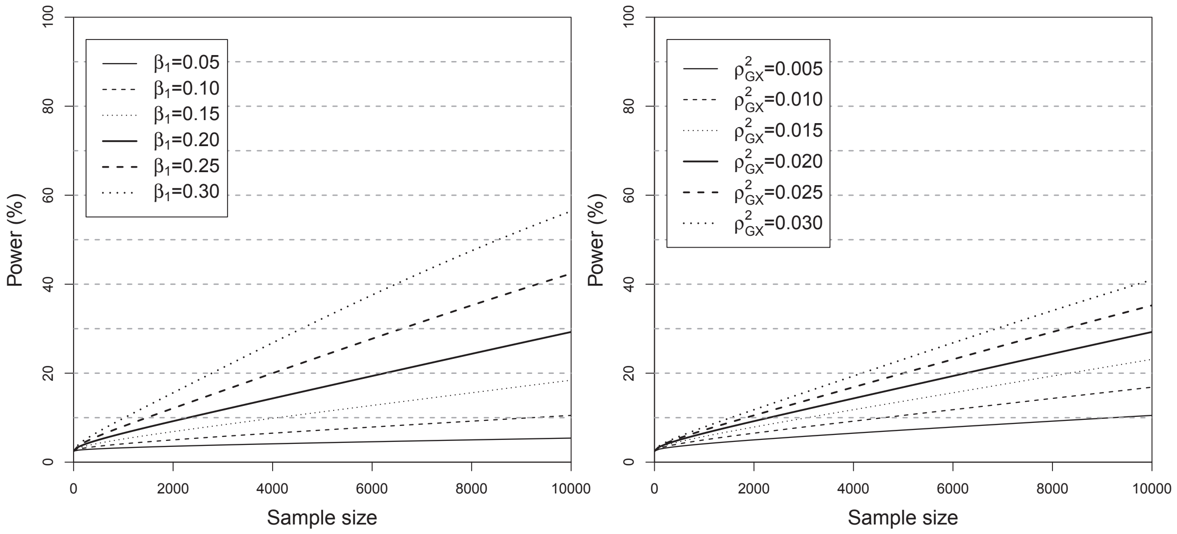

Web Figure A1: Power curves varying the sample size with binary outcome and a single instrumental variable: (left panel) for a fixed value of the IV strength ($\rho^2_{GX} = 0.02$) and different values of the size of the causal effect ($\beta_1 = 0.05, 0.1, \ldots, 0.3$); (right panel) for a fixed value of the causal effect ($\beta_1 = 0.2$) and varying the size of the IV strength ($\rho^2_{GX} = 0.005, 0.01, \ldots, 0.03$)
